# Transcriptome Sequencing of *Gracilariopsis lemaneiformis* to Analyze the Genes Related to Optically Active Phycoerythrin Synthesis

**DOI:** 10.1371/journal.pone.0170855

**Published:** 2017-01-30

**Authors:** Xiaoyun Huang, Xiaonan Zang, Fei Wu, Yuming Jin, Haitao Wang, Chang Liu, Yating Ding, Bangxiang He, Dongfang Xiao, Xinwei Song, Zhu Liu

**Affiliations:** Key Laboratory of Marine Genetics and Breeding, Ministry of Education, Ocean University of China, Qingdao, Shandong, China; Chinese Academy of Medical Sciences and Peking Union Medical College, CHINA

## Abstract

*Gracilariopsis lemaneiformis* (aka *Gracilaria lemaneiformis*) is a red macroalga rich in phycoerythrin, which can capture light efficiently and transfer it to photosystemⅡ. However, little is known about the synthesis of optically active phycoerythrinin in *G*. *lemaneiformis* at the molecular level. With the advent of high-throughput sequencing technology, analysis of genetic information for *G*. *lemaneiformis* by transcriptome sequencing is an effective means to get a deeper insight into the molecular mechanism of phycoerythrin synthesis. Illumina technology was employed to sequence the transcriptome of two strains of *G*. *lemaneiformis*- the wild type and a green-pigmented mutant. We obtained a total of 86915 assembled unigenes as a reference gene set, and 42884 unigenes were annotated in at least one public database. Taking the above transcriptome sequencing as a reference gene set, 4041 differentially expressed genes were screened to analyze and compare the gene expression profiles of the wild type and green mutant. By GO and KEGG pathway analysis, we concluded that three factors, including a reduction in the expression level of apo-phycoerythrin, an increase of chlorophyll light-harvesting complex synthesis, and reduction of phycoerythrobilin by competitive inhibition, caused the reduction of optically active phycoerythrin in the green-pigmented mutant.

## Introduction

The genus *Gracilaria* encompasses the greatest number of species in the family *Gracilariaceae*, with the majority of them being reported from warm-water and tropical regions. *Gracilariopsis*spp. are widely distributed around the world and are important economic macroalgae, as they are utilized for agar extraction and may play an important role in bioremediation [1, 2, 3, 4, 5]. *G*. *lemaneiformis* is the principal species for seaweed cultivation in China, used as a nutritious food source rich in beneficial proteins, minerals and trace elements [6]. Moreover, the majority of phycobiliproteins in *G*. *lemaneiformis* is phycoerythrin, as identified by fluorescence spectroscopy. Phycoerythrin is a bright red, fluorescent and water-soluble light harvesting pigment, commonly found in red algae. Phycoerythrin has a variety of characteristics, including solubility in water, antioxidant property, anti-tumor activity and so on, which promotes use of phycoerythrin in a wide range of applications in the food, cosmetic, clinical diagnostics, immunochemistry, biological engineering and other fields, as well as in research to develop other uses. The chromophore–protein complex in PBPs shows structural flexibility in response to environmental changes [7,8,9]. The green mutant of *G*. *lemaneiformis* has a completely different color, resembling green algae, along with changes of its spectral characteristics, which appear to result from the change in phycobiliprotein composition, especially the percentage of phycoerythrin. The green *G*. *lemaneiformis* pigmentation mutant could be useful for phycobiliprotein research, because the molecular mechanism of phycoerythrin biosynthesis in *G*.*lemaneiformis* is little known. Thus, in this study, next generation sequencing technology was used to help us analyze the differentially-expressed genes between the wild type and the pigment mutant at the level of transcription, with the objective of getting an improved understanding of the biosynthesis of phycoerythrin having optical activity.

In recent years, high-throughput sequencing technology has emerged as a powerful and cost-efficient tool for transcriptome analysis [10]. Transcriptome sequencing is not dependent on prior knowledge of gene sequences, and the *de novo* assembly of transcripts in non-model plant species that lack a reference genome is possible [11]. Transcriptome sequencing is an efficient way to measure transcriptome composition, obtain RNA expression patterns, and discover new genes [12].

In the present study, Illumina technology was used to sequence the transcriptome of *G*. *lemaneiformis* and a green pigment mutant. After merging effective reads of two samples, Trinity software was exploited to assemble these short reads into contigs, and then contigs were assembled into scaffolds. Finally, the repository of the *G*. *lemaneiformis* transcriptome was established. Taking these unigenes as reference genes; RNA-seq (quantification) technology was used to analyze the differentially transcribed genes in the green mutant with the objective of gaining a better understanding of the phycoerythrin synthesis and activity in *G*. *lemaneiformis*. This dataset will serve as a public information platform for gene expression, genomics, and functional genomics in *G*. *lemaneiformis*. The assembled and annotated transcriptome sequences will provide a valuable resource for future genetic and genomic research in *G*. *lemaneiformis* and closely related species.

## Materials and Methods

### Algal strains

The green mutant was discovered among wild-type fronds of *Gracilariopsis lemaneiformis* on the Nanao Island. The morphology and growth of the green mutant is almost the same as the normal group. After collection from Nanao island (Coastal Experimental Station of Shantou University in Nanao Island) in October, the green mutant and the wild type of *G*. *lemaneiformis* were thoroughly rinsed with sterilized sea water and cultured in 5L flasks containing 2L sterilized modified f/2 medium supplemented with 100 μM NaNO_3_ and 10 μM NaH_2_PO_4_·H_2_O, under a light intensity of 50 μmol photons m^-2^s^-1^ for a 12:12 light/dark period at 23±1°C. The medium was changed every three days. After a period of cultivation, the algae were frozen in liquid nitrogen and stored at -80°C for RNA extraction.

### Total RNA extraction and library construction

Total RNA of the wild type and the green mutant was extracted from the freshly frozen tissue samples by using TIANGEN reagent (Novogene), according to the manufacturer's instructions. Then, these RNA samples were tested to ensure the quality met the standard for library construction with regard to four aspects. First, the levels of degradation and contamination of RNA samples were checked by agarose gel electrophoresis. Second, RNA purity was checked by Nanodrop to confirm that it was of sufficient quality with an OD 260/280 ratio value near 2. Third, the concentration of RNA samples was accurately quantified by Qubit. Fourth, Agilent 2100 was used to check the integrity of RNA samples. When the RNA quality parameters met the requirements, the cDNA library could be constructed.

Magnetic beads with oligo(dT) were used to isolate eukaryotic mRNA from the total RNA. Subsequently, fragmentation buffer was added to chop the mRNA into short fragments, which were then used as the template for single-stranded cDNA synthesis by adding 6-bp random hexamers. Then, the second-strand cDNA synthesis was performed by adding buffer, dNTPs, DNA polymerase I and RNase H. After purification of the double-stranded cDNA using AMPure XP beads, the ends were repaired and poly (A) was linked to the 3’-cDNA. Sequencing adapters, which contained a recognition site, were added to the end of poly(A). After amplifying the double-stranded cDNA, the PCR amplification product was purified by AMPure XP beads. Finally, the cDNA library was constructed.

After construction of the cDNA library, the preliminary initial quantitation of the library was performed by Qubit 2.0. Then, the cDNA concentration was diluted to 1.5ng/μl. Subsequently, insert size was checked by Agilent 2100. After ascertaining that the insert size met the requirement, the effective concentration of the library was accurately quantified by Q-PCR (Effective concentration of library >2 nM) to guarantee the quality of the library.

### Illumina sequencing, quality controls and *de novo* assembly

The pools for wild type and the green mutant were used to generate two paired-end cDNA libraries, which were then sequenced using Illumina HiSeq^TM^2500 and MiSeq^TM^ sequencers. The raw data were obtained and the base quality was tested using Qphred software. Q_10_, Q_20_, Q_30_, Q_40_ represent an accuracy of 90%, 99%, 99.9%, 99.99%, respectively. Then, the low-quality reads were filtered out, (i.e., reads containing primer/adaptor sequences, reads in which the proportion of N (i.e. undetermined bases) were greater than 10%, reads with more than 50% proportion of bases with Qphred**≤**5, and so on) and the remaining clean reads were retained. The raw data were submitted to NCBI SRA with accession number SRP095068.

Transcriptome *de novo* assembly was carried out using Trinity [11]. Clean reads were virtually chopped to form a k-mer (k = 25) library by the Trinity software, to construct a dictionary of k-mers. Then, the k-mer reads with a certain length of overlap were combined to extend the sequence until they could not be further extended. These final extended sequences are called contigs. Contigs were assembled into components based on their overlap relationships. Then pair-end reads were re-used to fill the gaps in the components in order to obtain the unigenes. Finally, we got the assembled results of TRINITY. fasta.

### Gene function annotation, classification and CDS forecast

To obtain comprehensive information about gene function, unigene sequences were annotated by blast X and analyzed using the NR, NT, Swiss-prot (e-value = 1e^-5^), Pfam (e-value = 0.01), KOG (e-value = 1e^-3^), KEGG (e-value = 1e^-10^), and GO (e-value = 1e^-6^) database resources. On the basis of gene function annotation, genes can be classified into three categories: BP (biological process), CC (cellular component) and MF (molecular function) by Gene Ontology. Using the KOG database, unigenes can be divided into 26 groups. Unigenes can be mapped into different metabolic pathways by using KEGG. If the results of one database conflict with another one, the priority order of the databases should be NR, GO, KOG, Swiss-prot, NT.

ORF information was extracted according to the annotated unigene sequences directly from databases. Then, the coding region was translated into amino acid sequences (5'-3'direction).When the unigene sequences failed to match to the protein databases, ESTscan (3.0.3) software was applied to predict the ORF of these sequences. Finally, the nucleic acid sequences and the amino acid sequences encoded by them were annotated and could be applied for further study.

### GO and KEGG enrichment analysis of DEGs

Pathway enrichment analysis was used to identify significantly enriched metabolic pathways or signal transduction pathways of DEGs by comparing them with the whole genome background. Screening Threshold was qvalue <0.005 and | log_2_ fold-change| > 1 (Log_2_ fold-change = log_2_(Read count of Gg/Read count of Gr), Gr stands for the sample of the wild type while Gg stands for the sample of green mutant.). For the differentially expressed genes, log_2_ fold-change >0 means DEGs of the pigment mutant were up-regulated, log_2_ fold-change < 0 means DGEs of the pigment mutant were down-regulated compared to the wild type. After calculating the p-value, multiple hypothesis testing was carried out to try to reduce false positives. The smaller the p-value is, the larger the difference.

DEGs were mapped to the term types of the Gene Ontology database (http://www.geneontology.org/), and the numbers of DEGs were calculated in every term type. The significantly enriched terms were found by comparing to the whole genome background [13]. This method of enrichment analysis is termed GOseq [14].

KEGG is the main public database for pathway determination [15]. KEGG enrichment can identify the principal metabolic pathways and signal transduction pathways of DEGs. The KEGG significant enrichment was analyzed by the hypergeometric test to look for the significant enrichment pathway of DEGs compared to the all annotated genes by using KEGG Pathway as a unit. Computational formula of the analysis is:

p=1−∑i=0m−1(Mi)(N−Mn−i)(Nn)

N stands for the number of genes that can be annotated by pathways, and n is the number of DEGs in N. M is the number of genes annotated to a certain pathway among all the genes, and m is the number of DEGs in M. Those pathways with FDR (false discovery rate) ≤ 0.05 were defined as the significant enrichment pathways in DEGs.

### Gene validation and expression analysis

Based on the transcriptome result, genes encoding apo-phycoerythrin were chosen for validation of the transcriptome result, and study of phycoerythrin biosynthesis based on the differential expression between the green mutant and the wild-type using qRT-PCR. Total RNA was extracted from the wild type and the green mutant cultured in a light intensity of 54 μmol m^-2^ s^-1^ for 12h everyday for one week. The algae were powdered in liquid nitrogen, followed by RNA extraction using RNA extraction kits (Takara). The synthesis of cDNA was performed using a PrimeScript II 1st Strand cDNA Synthesis Kit (TaKaRa). Oligo dT primer and random 6-mer primers were both used as the RT-primers.

Real-Time qRT-PCR was performed using a SYBR Premix Ex Taq™ II Kit (TaKaRa) with a 20 μl reaction system containing 10.4μl 2×SYBR green Mastermix (TaKaRa), 0.3μl forward and reverse primers, 1 μl cDNA and 8 μl ddH_2_O. Reaction steps followed the following program: 95°C for 30 s, followed by 40 cycles of 95°C for 5 s, 55°C for 20 s, 72°C for 20 s and reading fluorescence signal, followed by 1 cycle of 95°Cfor 15 s, 60°C for 60s, 95°C for 15 s.

### Fluorescence spectrophotometer

First, equal thallus masses of the wild-type and the green pigment mutant were weighed and ground with liquid nitrogen in a mortar. Then, the powder was transferred into a 10 ml EP tube, 3 ml of 0.1mol/l PBS were added and the samples were sonicated at intervals for 6 minutes (repeated cycles of sonication for 2.5 seconds, pause for 5 seconds, for a total of 6 minutes). The mixture was centrifuged for 10 minutes at 10000r/min. Then 1 ml supernatant liquid was added into the cuvette to measure the fluorescence intensity of the characteristic peaks for phycoerythrin and chlorophyll.

## Results

### Short-read *de novo* sequencing and assembly

By sequencing the transcriptome of *G*. *lemaneiformis*, 30,450,973 and 28,306,016 raw reads of the wild type and the green mutant were obtained, respectively. To guarantee the quality of data used for the analyses, stringent parameters were used to measure the quality of reads, and all reads with undetermined information greater than 10% were removed. After this filtering, 29,217,420 and 27,230,252 clean reads of the wild type and green mutant were generated, with 94.97% and 95.14% bases showing quality greater than Q20, while the GC percentage was 52.42% and 52.83% respectively. Then, the k-mer (k = 25) technique was used to measure the nucleotide sequence of reads.

**Fig 1:**
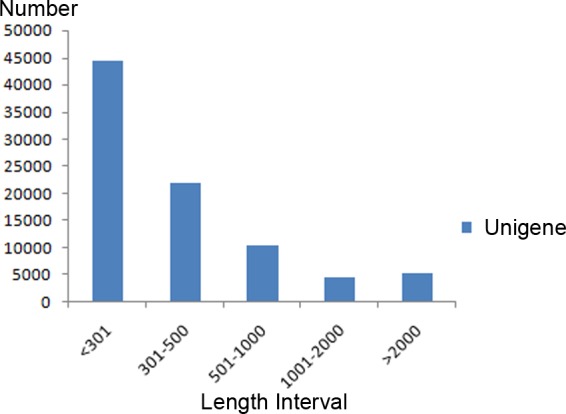
The length distribution of unigenes. The vertical axis represents the number of unigenes, and horizontal axis gives the length interval of the unigenes.

As a no-reference genome project, these clean reads were assembled into contigs using overlap information until the contigs could not be further extended. After a series of subsequent steps, 86915unigenes (>200 bp) were generated with N50 of 1481 bp and N90 of 245 bp. The size of the unigenes varied from 201 bp to 38052 bp with a mean length of 679 bp, and a total length of 59,051,626 bp. The length distribution of the unigenes is given in Fig 1.

### Function annotation

To acquire the comprehensive genetic information of *G*. *lemaneiformis*, the unigenes were blasted against the NR, NT, KO, SwissProt, PFAM, KOG, GO, KEGG database resources to identity the functions of all of the unigene sequences.

All of the unigenes were annotated to genes having known functions in the indicated databases based on the sequences with greatest similarity. Among these, 42884 unigenes were annotated in at least one database, 2070 unigenes were annotated in all of the databases (Table 1). In total, 36036 unigenes (41.46% of all unigenes) were aligned to the NR database with an e-value < 1e^-5^.

**Table 1:** Summary of the function annotation results for *G*. *lemaneiformis* unigenes in public protein databases.

	Number of Unigenes	(%)
Annotated in NR	36036	41.46
Annotated in NT	3544	4.07
Annotated in KO	12279	14.12
Annotated in SwissProt	18818	21.65
Annotated in PFAM	26822	30.86
Annotated in GO	32194	37.04
Annotated in KOG	14996	17.25
Annotated in all Databases	2070	2.38
Annotated in at least one Database	42884	49.34
Total Unigenes	86915	100

**Fig 2:**
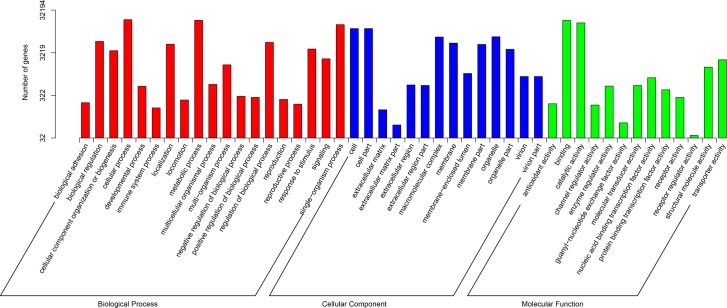
Gene function classification of all annotated unigenes by Gene Ontology. The vertical axis represents the number of unigenes, and horizontal axis gives the specific GO sub-categories.

A total of 32194 (37.04%) unigenes were annotated successfully by GO annotation. These annotated unigenes were classified into the next terms of three ontologies: BP (biological process), CC (cellular component) and MF (molecular function). The distribution of unigenes is shown in Fig 2. In many cases, the same unigene was assigned to multiple terms. Within the cellular component category, the majority of genes were assigned into “Cell” (11882, 36.91%) and “Cell Part” (11877, 36.89%). For the molecular function, most of genes were involved in “Catalytic Activity” (16154, 50.18%) and “Binding” (18384, 57.10%). Among the "Biological Process", a high percentage of genes were classified into “Cellular Process” (19233, 59.74%), “Metabolic Process” (18551, 57.62%) and “Single-organism Process” (14679,45.60%).The greatest number of annotated unigenes were involved in "Biological Process".

**Fig 3:**
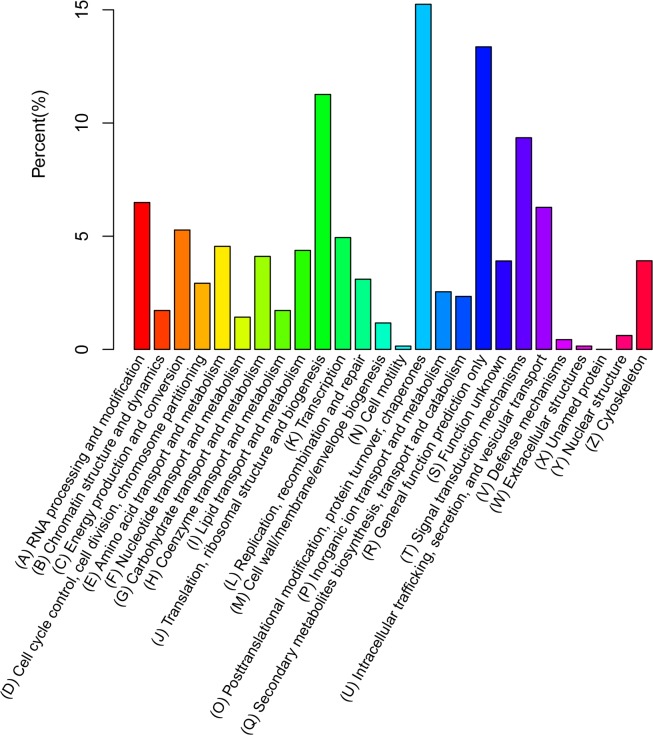
KOG distribution of the annotated unigenes. The y-axis represents the percent (%) of unigenes classified into the specific categories.

The unigenes was searched against the KOG database in order to predict and classify their possible functions. A total of 14996 unigenes were annotated successfully and classified into 26 groups by KOG annotation (Fig 3). Among the 26 KOG categories, “Posttranslational Modification", "Protein Turnover", and "Chaperones” account for the largest proportion of unigenes (2286 unigenes, 15.24%). The second largest group of unigenes were in “Translation", and "Ribosomal Structure and Biogenesis”(1689 unigenes, 11.26%), followed by “Signal Transduction Mechanisms”(1402 unigenes, 9.35%), “RNA Processing and Modification” (973 unigenes, 6.49%), “Intracellular Trafficking, Secretion, and Vesicular Transport” (941 unigenes, 6.28%).The smallest groups were “Cell Motility” and “Extracellular Structures” (22 unigenes, 0.15%).

**Fig 4:**
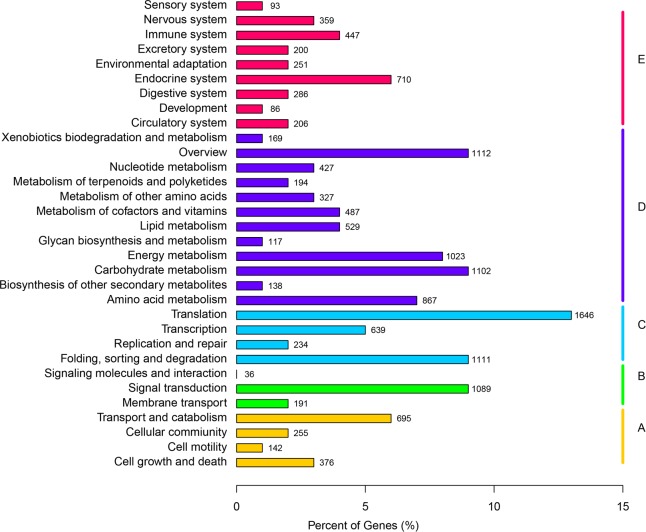
KEGG classification. Y-axis lists the various metabolic pathways, while the x-axis gives the percent of genes in the pathways. The KEGG pathway categories can be divided into five groups: A. Cellular Processes, B. Environmental Information Processing, C. Genetic Information Processing, D. Metabolism, E. Organismal Systems.

Based on the KO annotation results, all of the 12279 annotated unigenes could be classified into 268 pathways at the level of "Pathway Hierarchy 2" on the basis of their participation in KEGG metabolic pathways. KEGG pathways can be divided into five branches: "Cellular Processes", "Environmental Information Processing", "Genetic Information Processing", "Metabolism", and "Organismal Systems". Most of the unigenes were involved in the "Translation Process" of "Genetic Information Processing"(1646unigenes, 13.41%), followed by "Folding, Sorting and Degradation" (1111, 9.05%), "Carbohydrate Metabolism" (1102, 8.97%), " Signal Transduction" (1089, 8.87%), and "Energy Metabolism" (1023, 8.33%) (Fig 4).

Based on the results above, a large number of unigenes were found to match known proteins in the four public databases, which provided detailed functional information. This function analysis is essential to get a deeper understanding of the gene expression profiles in *G*. *lemaneiformis*. At the same time, these annotations contribute to identifying unigenes and the pathway involved in the synthesis of phycoerythrin.

### DEG and DEG GO enrichment

In the present study, the primary goal of transcriptome sequencing was to compare gene transcriptional levels between the wild type and the green-pigmented mutant. A total of 4041 differentially expressed genes (5.6% of all detected genes) were identified. All these DEGs were screened by “the absolute value of log_2_ Ratio ≥ 1 and FDR ≤ 0.001” between the two libraries. In the green mutant library, 3726 DEGs were up-regulated and 315 DEGs were down-regulated compared to the wild type.

**Fig 5:**
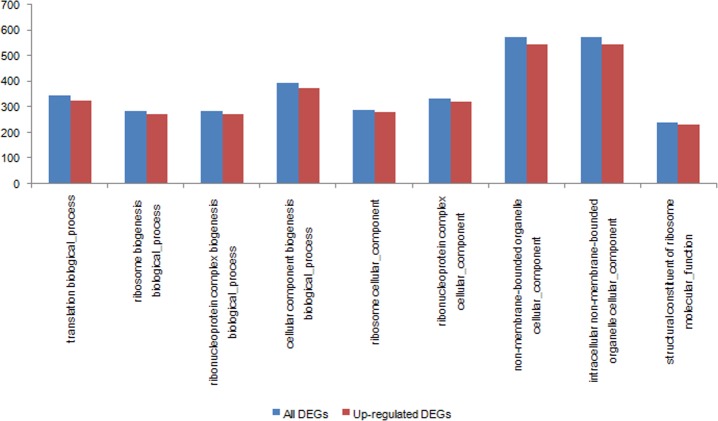
The comparison of GO enrichment between all DEGs and up-regulated DEGs.

In order to get a better understanding about the biological function of these DEGs, GOseq was used to divide these DEGs into different GO terms. A total of 3239 DEGs were classified by GO functional annotation. In general, "non-membrane-bounded organelle cellular component" (GO:0043228) and "intracellular non-membrane-bounded organelle cellular component" (GO:0043232) account for the largest proportion of DEGs (572 DEGs, 17.66%), followed by "cellular component biogenesis biological process" (GO:0044085) (393 DEGs, 12.13%), "translation biological process" (GO:0006412) (344 DEGs, 10.62%), "ribonucleoprotein complex cellular component" (GO:0030529) (335 DEGs, 10.34%), "ribosome cellular component" (GO:0005840) (289 DEGs, 8.92%), "ribonucleoprotein complex biogenesis biological process" (GO:0022613) (286 DEGs, 8.83%), "ribosome biogenesis biological process" (GO:0042254) (284 DEGs, 8.77%), "structural constituent of ribosome molecular function" (GO:0003735) (241 DEGs, 7.44%) (Fig 5). The results indicate that at least 54.92% of the DEGs directly participated in the process of protein synthesis by affecting the structure of the ribosome and the protein translation process.

**Fig 6:**
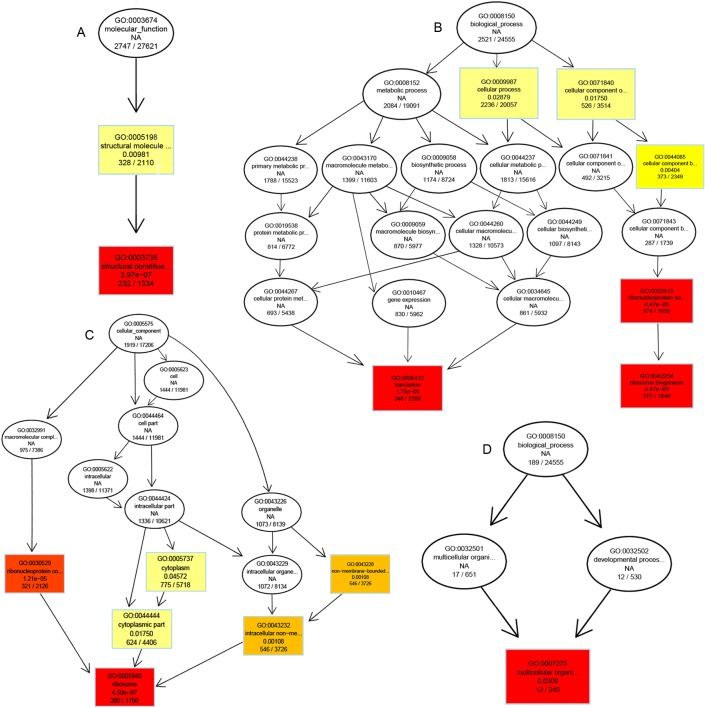
Biological Process, Cellular Component and Molecular Function network with significantly enriched GO terms in DGEs between wild type and green pigment mutant. (A) Up-regulated DEGs of Molecular Function in green pigment mutant compared to control. (B) Up-regulated DEGs of Biological Process in green pigment mutant compared to wild type. (C) Up-regulated DEGs of Cellular Component in green pigment mutant compared to wild type. (D) Down-regulated DEGs of Biological Process in green pigment mutant compared to wild type. The colored nodes represent the level of enrichment, and the deeper the color, the higher the enriched level.

With the aim of getting detailed information of DEGs, these DEGs were divided into two groups (up- and down-regulated DEGs). Up-regulated DEGs were compared with the total DEGs. The results indicated that DEGs with enriched GO terms were mainly up-regulated genes, and all of these GO terms’ "Corrected_pValues" were less than 0.05. In the biological process category, the end nodes of the GO terms for up-regulated DEGs were mainly enriched in “translation” and “ribosome biogenesis biological process”, and down-regulated DEGs were mostly included in “multicellular organismal development”. In the "Cellular Component" GO network analysis, the significantly enriched GO terms focus on “Ribosome Cellular Component”. In the Molecular Function category, the GO terms of up-regulated DEGs were enriched in “Structural Constituent of Ribosome” (Fig 6).

In conclusion, the results of the GO functional enrichment analyses of DEGs indicated that the biggest difference of all DEGs between the wild type and the green pigment mutant was for the unigenes related to biological process of protein and the structure of ribosome. The results also indicated that the significantly up-regulated ribosomal subunit composition may increase the synthesis of protein.

### KEGG Pathway analysis and detection of candidate genes

Pathway-based analysis plays an important role in getting a further understanding of the interactions among genes in the same pathway. A total of 4041 DEGs were analyzed against the KEGG databases. These DEGs were enriched into 294 pathways by the hypergeometric statistical test. The top 20 comprehensive, up-regulated and down-regulated DEG KEGG enriched pathways were identified. Generally, the largest number of DEGs (141) was enriched in the pathway of Ribosome, among these, 140 DEGs were up-regulated and 1 DEG was down-regulated. The results indirectly indicated that in the green pigment mutant, the function of protein synthesis increased significantly.

**Fig 7:**
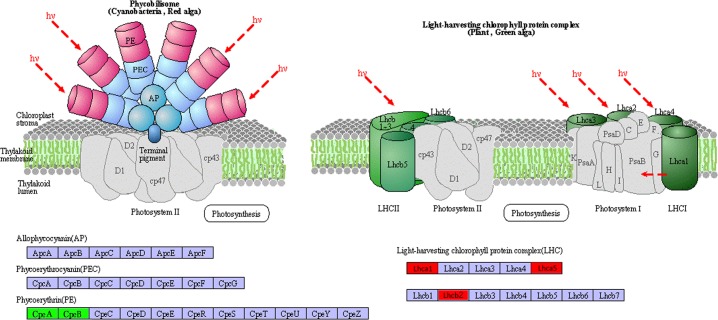
Apo-phycoerythrin and light-harvesting chlorophyll protein complex function-related KEGG pathway. CpeA, and CpeB represent theα and β subunit of apo-phycoerythrin, respectively. Lhc represents the light-harvesting chlorophyll protein complex, the green color represents the down-regulated DEGs, red color represents the up-regulated DEGs.

**Fig 8:**
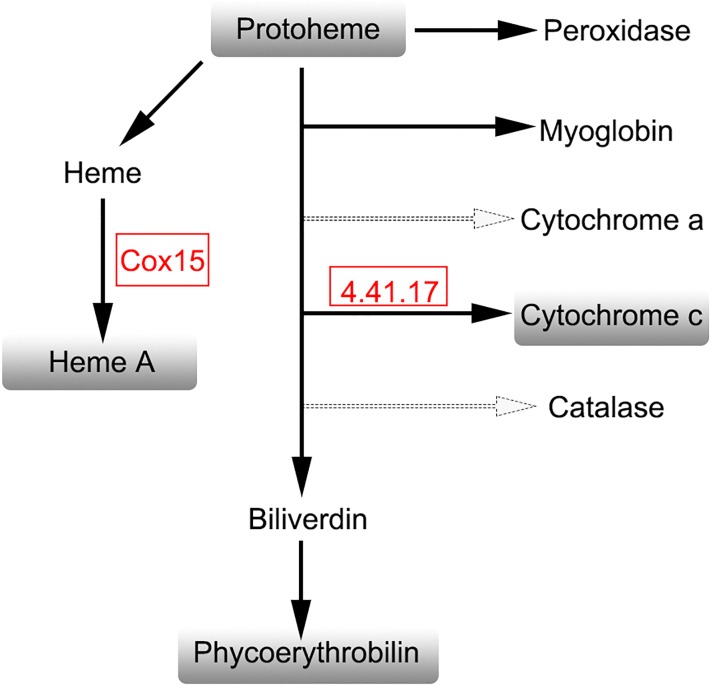
The metabolic pathway of phycoerythrobilin (porphyrin and chlorophyll metabolism). Red box represents up-regulated DEGs. The dashed lines represent omitted intermediate steps. 4.4.1.17 represents the cytochrome c oxidase assembly protein subunit 15.

Apo-phycoerythrin was identified as a down-regulated DEG, as shown in Fig 7, the expression of the green mutant's light-harvesting chlorophyll protein complex increased significantly. Meanwhile, the DGEs related to the synthesis of porphyrin and chlorophyll metabolism were enriched (Fig 8). For porphyrin and chlorophyll metabolism, a large number of genes were highly expressed in the green mutant compared to the wild type of *G*. *lemaneiformis*. Interestingly, these genes are related to the synthesis of biliverdin, which is a precursor of phycoerythrin. Peroxidase, myoglobin, cytochrome a, cytochrome c and catalase compete with biliverdin for heme. The up-regulation of COX15 and cytochrome c oxidase assembly protein subunit 15 (4.4.1.17) might competitively decrease the synthesis of biliverdin. For phycoerythrin to be optically active, apo-phycoerythrin must be bound to phycoerythrobilin. In this study, the transcription level of apo-phycoerythrin was decreased, and the synthesis of phycoerythrobilin was competitively inhibited in the green mutant. At the same time, the transcription level of the light-harvesting chlorophyll protein complex was increased. These changes in pigment composition are clearly responsible for the green color of the mutant.

### Analysis of the results of Real-time quantitative PCR

**Fig 9:**
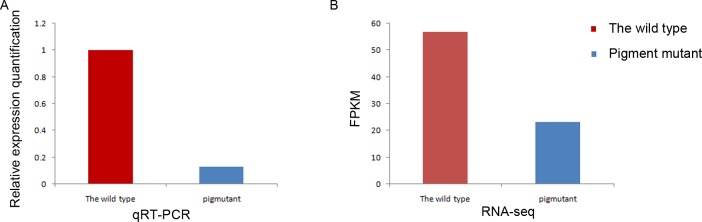
Differential expression analysis of apo-phycoerythrin (c13260-g1) between wild type and the green mutant. (A) The result of qRT-PCR. (B)The result of RNA-seq. FPKM represents the expected number of fragments per kilobase of transcript sequence per millions of base pairs sequenced, which provided a favorable reliability for the quantitative analysis of DEG.

To validate the sequencing results obtained by RNA-seq, real-time PCR was performed on the apo-phycoerythrin gene related to the biosynthesis of phycoerythrin. The result showed that the expression of apo-phycoerythrin was much higher in the wild type than the pigment mutant, which is similar to the RNA-seq data (Fig 9). The result verified the reliability of the transcription sequencing results.

### Fluorescence spectra analysis of phycoerythrin

**Fig 10:**
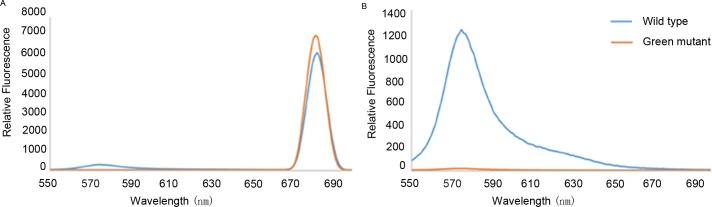
Excitation spectra for phycoerythrin and chlorophyll in the wild type and green mutant. (A) Fluorescence emission at 340 nm. (B) Fluorescence emission at 480 nm. Phycoerythrin absorbs maximally around 580 nm, while Chlorophyll absorbs maximally around 680 nm. Characteristic peaks at different wavelengths can show the proportion between phycoerythrin and chlorophyll in the wild type and green mutant.

To validate the analysis results of the KEGG pathway analysis, a fluorescence spectrophotometer was used to analyze fluorescence intensity of phycoerythrin at the characteristic peaks, for green mutant and wild type. We set the wavelength of emission light at 340nm and 480 nm, respectively (Fig 10). Phycoerythrin absorbs maximally around 580nm, while Chlorophyll absorbs maximally around 680nm. Characteristic peaks at different wavelength can show the proportion between phycoerythrin and chlorophyll in the wild type and green mutant. When fluorescence emission was at 340nm, the characteristic peak of the wild type was lower than the green mutant, indicating there was a higher level of chlorophyll in the green mutant. When fluorescence emission was at 480nm, the characteristic peak of the wild type was very high while there was no characteristic peak in the green mutant, indicating that the level of phycoerythrin in the wild type is much greater than in the green mutant.

## Discussion

RNA-Seq has proved useful for the detection of gene expression, the discovery of novel transcripts, and the identification of differentially expressed genes. Here, second generation sequencing technology was used to analyze the transcriptome of *G*. *lemaneiformis*, and DEG analysis was used to reveal the change of expression pattern in the green mutant.

Red algae contain phycobiliproteins in addition to chlorophyll a, chlorophyll b, carotene and lutein. Consequently, red algae have two light-harvesting systems including the peripheral antenna system consisting of phycobilisomes and the internal antenna system comprised of the chlorophyll protein complex. Phycobiliproteins are pigments composed of colorless apoproteins that bind several light-absorbing phycobilin chromophores [16]. Phycoerythrin, as the main antenna, plays an important role in normal *G*. *lemaneiformis* giving it a red color, when phycoerythrin is successfully synthesized and the phycoerythrobilin is attached to the apo-phycoerythrin.

By KEGG Pathway analysis, the transcriptional level of apo-phycoerythrin in the green mutants was significantly down-regulated, about 1.2 times, which may be the direct cause of the low level of phycoerythrin in green mutants. Meanwhile, in the porphyrin and chlorophyll metabolic pathway, cytochrome c heme-lyase, and cytochrome c oxidase assembly protein subunit 15, increased 4–6 times, which resulted in the higher level of cytochrome C and heme A. At the same time, the expression level of biliverdin decreased significantly by competitive inhibition, which negatively affected phycoerythrobilin synthesis. These results indicate a competitive relationship between phycoerythrobilin and chlorophyll synthesis in *G*. *lemaneiformis*, and also demonstrated the importance of phycoerythrobilin and phycoerythrin synthesis for the synthesis of phycoerythrin with optical activity.

In photosynthetic organisms, solar energy in the form of photons is gathered by light-harvesting complexes (LHC) and then transferred to reaction centers, where the excitation energy is converted into electrochemical energy by charge separation [17]. Concomitant with the decrease of the phycoerythrin level, the chlorophyll light-harvesting protein complexes, including Lhca1, Lhca5, Lhcb2 were up-regulated 4–8 times. Plants and microalgae respond to light quantity changes by either reducing (excess light) or increasing (limiting light) the cellular LHC level [18, 19, 20]. Therefore, the increase in light-harvesting protein may compensate for the decrease in the amount of light energy harvested due to the drop in the phycoerythrin level.

**Fig 11:**
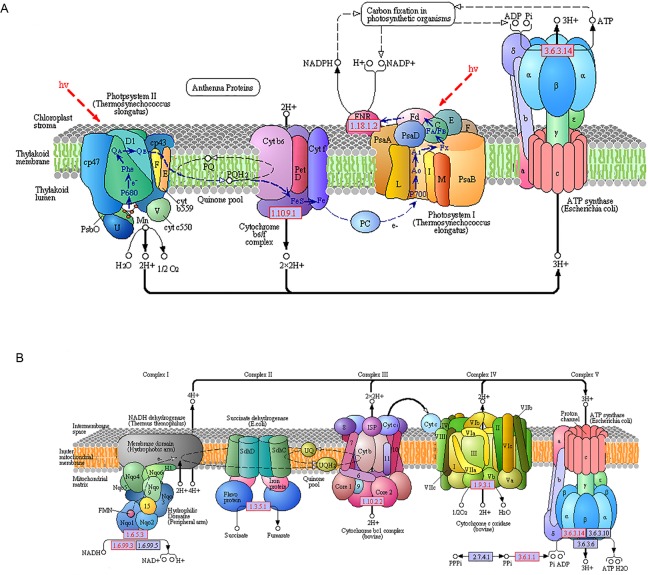
The pathway of photosynthesis and oxidative phosphorylation. (A) The pathway of photosynthesis. (B) The process of oxidative phosphorylation. Red numbered boxes indicate up-regulated genes in the green mutant as compared to the wild type.

The energy captured in an excited chlorophyll molecule can have several fates: it can be transferred to another pigment, used to drive the redox reactions of photosynthetic electron transfer (PET), dissipated thermally as heat, or re-emitted as fluorescence. Because these processes are in competition with each other, any one of them will be modified by changes in any of the other pathways [21]. In this study, the expression level of proteins related to photosynthetic electron transport in the green mutant increased significantly. In PSⅡ, the expression level of three protein subunits including PsbO, PsbS, PsbU increased 4–6 times, in the cytochrome b6-f complex, the Fe-S subunit increased 5.7 times, and ferredoxin—NADP+ reductase increased 5–8 times in PSⅠ. All of these up-regulated proteins play the role of electron transport carrier between PSⅠand PSⅡ. Therefore, the speed of electron transfer might accelerate between PSⅠ and PSⅡ. Based on the outcomes for the pathways for porphyrin and chlorophyll synthesis, we know that the expression quantity of cytochrome c increased, which is interesting because cytochrome c plays the role of electron transport carrier in the oxidative phosphorylation metabolic pathways. In addition, five subunits of cytochrome c oxidase and two subunits of cytochrome c reductase increased significantly, which might indicate that cytochrome c can be oxidized and reduced quickly, increasing the rate of electron transport (Fig 11). Electron transport always couples with ATP synthesis in photosynthesis and oxidative phosphorylation, thus the information gathered suggested that the synthesis of ATP was likely increased.

The expression level of ferredox in NADP reductase increased about 5–8 times, thus more NADPH would be available for synthesizing ATP to provide energy. The expression of NADH dehydrogenase, including Ndufs1, Ndufs2, Ndufs3, Ndufv1, Ndufv2, Nduf3v, Ndufa2, Ndufa6, Ndufa9, Ndufab1, also increased significantly, which would provide energy for synthesizing ATP directly. Three subunits of F-type ATPase α, β, γ, F-type H+-transporting ATPase subunit O and almost all of the subunits of V-type ATPase increased significantly, which could increase the rate of ATP synthesis. As a result, photophosphorylation increased significantly.

The GO functional enrichment analyses of DEGs suggested that the biggest difference of all DEGs between the wild type and the green pigment mutant was that the significantly up-regulated unigenes related to the biological process of protein and the structure of ribosome. Therefore, the results of DEG GO enrichment can be explained to a certain extent. On the one hand, photophosphorylation and oxidative phosphorylation provide the energy basis for the synthesis of ribosomes and protein. On the other hand, the enhancement of ribosome metabolic activity could allow the synthesis of more proteins related to energy metabolism. Increase of energy metabolism and material metabolism of the green mutant may help to compensate for the shortage of energy due to the reduction in the amount of phycoerythrin.

Through this comparative analysis between the wild type and the green-pigmented mutant, we identified the causes of the color variation in the mutant and identified the synthesis pathway of phycoerythrin. This study also set an important foundation for detecting potential genes related to the synthesis of phycoerythrin with optical activity. Additionally, *G*. *lemaneiformis* is able to increase the expression of the light-harvesting protein complexes to increase light capture when lacking phycoerythrin, which will provide an important base for studying the Lhc gene family.

## Conclusions

*G*. *lemaneiformis* is a suitable alga for studying the metabolic pathway of phycoerythrin, as phycoerythrin accounts for the largest proportion of pigments in the phycobiliprotein. The green mutant provides a good comparison for finding differential expression of genes for phycoerythrin synthesis. Transcriptome sequencing technology provides an effective means for analyzing the genetic information of this species. We have assembled 86915 unigenes using the Trinity *de novo* method, including identifying 42884 annotated unigenes in data bases, and screening 4041 differentially expressed genes. This study helps to provide a better understanding of the molecular mechanisms of photosystem regulation. The transcriptome dataset will take an active role in identifying genes related to photosynthesis in red algae, and it will also serve as a public information platform for studying gene expression and function in *G*. *lemaneiformis*.
